# Strategies for Coping with Time-Related and Productivity Challenges of Young People with Learning Disabilities and Attention-Deficit/Hyperactivity Disorder

**DOI:** 10.3390/children6020028

**Published:** 2019-02-13

**Authors:** Consuelo M. Kreider, Sharon Medina, Mackenzi R. Slamka

**Affiliations:** Department of Occupational Therapy, University of Florida, Gainesville, FL 32603, USA; sharonmedina@ufl.edu (S.M.); mslamka@ufl.edu (M.R.S.)

**Keywords:** self-management, neurodevelopmental, time management, college students, qualitative research, invisible disabilities, neurorehabilitation, executive functions

## Abstract

Learning disabilities (LD) and attention deficit hyperactivity disorder (ADHD) are characterized by neurological differences that result in difficulties meeting learning and productivity expectations. Young people with LD and ADHD experience difficulties in self-managing academic, social, daily living, and health/wellness demands. Students with LD/ADHD must work longer and harder than peers, which makes managing time and productivity a critical skill for school success. This study examined the strategies that college students with LD/ADHD used to overcome obstacles related to time and productivity within their everyday life contexts. A qualitative phenomenological design was used to examine the phenomenon of coping and productive-task performance through strategy use among 52 college students with LD/ADHD. Strategies classified as habit and routine use, reframing, and symptom-specific strategies were identified. Strategy use for addressing time-related and productivity challenges are multidimensional and entailed a mix of cognitive, behavioral, psychological, and socio-environmental strategies. Effective strategy use across life’s contexts was critical to self-managing as a young person with a chronic developmental condition within a college context. The findings provide a much-needed understanding of the multi-faceted challenges and solutions within young adult contexts that are important for guiding the development of interventions for young people with LD/ADHD.

## 1. Introduction

Learning disabilities refer to a highly prevalent and diverse group of disorders involving neurological differences that result in impaired learning and difficulties in information processing and executive functioning. These difficulties impact organization and time management abilities, which are needed for productivity within educational and daily life contexts. Within the educational system, “learning disabilities (LD)” is used as an umbrella term to categorize a group of conditions that affect a student’s abilities to read, write, listen, speak, reason, and/or do math [1]. It encompasses clinical conditions such as specific learning disorder, which entails a persistent pattern of difficulties in reading, writing, and/or mathematics [2]; auditory processing disorder; developmental language disorder; and developmental coordination disorder [3]. Attention-deficit/hyperactivity disorder (ADHD) is a highly prevalent condition that co-occurs with LD at a rate of almost 50% [4]. Attention-deficit/hyperactivity disorder is a neurodevelopmental condition that involves an ongoing pattern of inattention, impulsivity, and/or hyperactivity that interferes with the individual’s functioning and/or development [2]. Both LD and ADHD (LD/ADHD) are characterized by social skill deficits [5] and weaknesses in executive functioning of the brain [6,7,8]. Executive functioning difficulties impact executive attention, working memory, inhibition, mental flexibility, temporal processing, and emotional and behavioral self-regulation for those with LD/ADHD [7,8]. 

Although primarily considered childhood conditions, LD/ADHD are lifelong conditions whereby difficulties persist into adulthood [9,10], thus putting young people with these conditions at greater risk for negative adult outcomes. Adults with LD/ADHD experience higher risks for difficulties in nonacademic areas; they report higher rates of emotional difficulties (e.g., depression and anxiety), social problems, and difficulties in attaining and maintaining satisfying employment [11,12]. For young people transitioning to adulthood through college pathways, LD/ADHD-related difficulties create additional challenges in self-managing academic, social, daily living, and health/wellness demands [13]. Childhood interventions are critical for optimizing life course trajectories of young people with disabilities, especially interventions that can impact the child’s functioning across multiple contexts. As such, multidisciplinary perspectives are essential for meeting the multi-faceted needs of young people with LD/ADHD.

Learning disability and ADHD-related cognitive difficulties impact the mental processes used in planning and performing activities and tasks across contexts within the individual’s everyday life situations. Successful performance entails the ability to plan and carry-out roles, routines, and tasks within everyday life situations in response to expectations and demands [14]. Young people transitioning to adult roles and contexts are expected to develop and meet personal goals, work toward establishing a career, and build healthy interpersonal relationships and daily routines. For those with LD/ADHD, especially those pursuing post-secondary education, persisting cognitive deficits can pose real challenges to the development and implementation of executive skills needed for college success [15]. Emerging adults with LD/ADHD are challenged in managing daily life and the time and tasks related to accomplishing daily goals [16]. Such challenges are contributed to by the inconsistent use of executive skills by individuals with LD/ADHD [17].

For college students with LD/ADHD, difficulties with planning and managing academically related time are compounded by slower learning and slower production of academic work [17]. These students must work longer and harder than their peers in order to compensate [17], which makes skills and strategies used for managing time and productivity critical to college success. A strategy is a tool, plan, or method used to enhance information processing, efficiency, and/or performance in order to achieve success in a task [18,19]. For those with LD/ADHD, obstacles to effective strategy use stem from difficulty with automatically generalizing learned strategies and skills to new situations [20]. As such, the ability to generate, adjust, and consistently apply strategies that support activity and task performance across the range of life’s situations are of critical importance for individuals with LD/ADHD.

Empirically informed compensation strategies for LD/ADHD-related challenges center on academic compensations, such as extended time for tests, and on strategies for overcoming academic-related obstacles, such as notetaking strategies [21]. However, there remains a substantial lack of understanding regarding the approaches and strategies used by young people with LD/ADHD for coping with time-related and productivity challenges experienced beyond the classroom context. Moreover, little research exists that provides guidance in developing strategies for coping with LD/ADHD-related difficulties in performance within everyday life contexts, with even less guidance available specific to the contexts of emerging adulthood. This study examined, in-depth, the strategies that young people in college with LD/ADHD used to overcome obstacles related to time and productivity within their everyday life contexts. 

## 2. Materials and Methods

A qualitative phenomenological design was used to examine the phenomenon of coping and the productive-task performance through strategy use by college students with LD/ADHD [22]. This paper reports on a secondary data analysis. Data were transcripts from group discussions and individual interviews that were collected as part of a larger study, which developed and tested a campus-based model of holistic LD/ADHD supports [23]. The data were originally collected for the purpose of gaining actionable insights into the participants’ needs and experiences as emerging young adults with LD/ADHD within a college environment.

Study activities took place at the University of Florida, which is a large research-intensive university in the United States. Undergraduates were eligible for study participation if they were registered with the campus disability office to receive academic accommodations related to LD. The campus disability office categorized LD to include neurodevelopmental disorders of reading (i.e., dyslexia), math (i.e., dyscalculia), writing (i.e., dysgraphia), coordination (i.e., developmental coordination disorder), auditory processing, language processing, and/or ADHD. Inclusion criteria also required undergraduates to be available for two academic years and enrolled in a science, technology, engineering, or mathematics field of study. The presence of additional health conditions did not exclude qualifying participants from the study. Recruitment was achieved through the use of campus and disability office listservs, flyers posted on campus, the project website, and word-of-mouth. Participants were enrolled for four consecutive non-summer semesters. They received mentorship from a graduate student in a similar field of study and engaged in monthly group meetings where psychoeducational content providing LD/ADHD-related knowledge and facilitated group discussions were provided. Study procedures were approved by the University of Florida Gainesville Health Science Center Institutional Review Board; procedures were originally approved on 20 August 2012 (IRB# 465-2012) with the most recent annual approval received 21 December 2018. All undergraduate participants provided written informed consent prior to engaging in study activities.

Data regarding participant characteristics were collected upon enrollment via a secure online survey. The survey included demographic and symptom survey questions that were developed for the study. Participants rated the severity of targeted symptoms using a digital visual analog scale which was anchored by “never” (numerical score of 0) and “always” (numerical score of 100). The digital visual analog scale is a valid, reliable, and responsive patient-rating measure of perceived symptom severity [24]. The online survey platform allowed respondents to rate symptoms by moving a vertical slider mark across a horizontal scale. Descriptive statistics were used to understand participants’ demographics and symptom ratings.

The qualitative dataset included transcripts from individual interviews (*n* = 15) conducted during the first year of the study and from monthly group meetings/discussions (*n* = 30) collected over four years. Audio recordings were professionally transcribed verbatim and checked by researchers for accuracy. A conceptual thematic analysis of the qualitative data was conducted. Structural coding was first used to identify textual passages containing descriptors of temporal and productivity challenges and strategies used. Process coding was then used to label salient textual data that depicted participants’ challenges, actions/interactions, and associated emotions. Process coding entails the identification and classification of participant actions [25]. Codes were then reduced, and conceptual categories were identified through the use of axial coding. Axial coding entails the identification of linkages among concepts through a process of inductive reasoning and constant comparison of the data to emerging conceptualizations [25]. Data analysis was supported by the use of the NVivo Software (QSR International Pty Ltd. Version 10, 2012.) for qualitative data analysis. Analytic rigor was enhanced through the use of multiple coders, regularly scheduled discussions of emerging findings amongst members of the research team, peer debriefing, and researchers’ extended interactions with study participants.

## 3. Results

### 3.1. Participants

Fifty-two undergraduates with LD/ADHD, ages 21.2 ± 3.5 years, were enrolled in the study. Twenty-two (42%) participants reported an LD, 18 (35%) reported ADHD, and 12 (23%) reported co-occurring LD and ADHD. Twenty (38%) participants reported having an additional mental health condition, of which 4 identified as having autism spectrum disorder; 12 reported clinical anxiety, depression, and/or obsessive-compulsive disorder; and 4 did not specify the additional mental health condition. Table 1 delineates the participant demographics, and Table 2 describes the participant ratings of LD/ADHD-related symptoms and challenges.

### 3.2. Strategies

Strategies for managing LD/ADHD-related temporal and productivity challenges were used across the multiple contexts of participants’ lives as emerging adults. Contexts fell within the domains of academics, daily life, career, health, and social interactions. Table 3 outlines the strategies used by participants.

#### 3.2.1. Habits and Routines

Habits and routines describe participants’ everyday practices that provide structure to their day and promote productive living across various contexts such as academic, career, daily, and social life. Key habits and routines include having (1) a structured and productive morning routine, (2) a planning system, (3) prioritization strategies, and (4) reminder systems. These strategies were important for helping participants manage schedules, goals, tasks, and expectations in both the immediate and distant future. For study participants, habits and routines served to reduce cognitive load and improved efficiency and/or the accuracy of task performances.

Some participants spoke of using a highly structured and productive morning routine in order to free up both time and mental energy for the remainder of their day. Participants described morning productivity that centered on daily and healthy living tasks, such as laundry and meal preparation. For these participants, focusing on such tasks early in the day enabled them to focus on academic and career development demands throughout the day. Participants who used this strategy spoke of intensely protecting the maintenance of a structured and productive morning routine. For example, one participant described waking up between four and five o’clock each morning to complete all daily living chores (e.g., laundry, meal prep) and to exercise before leaving for her first morning class. As shared by another, “I have four animals... I check on the chinchilla and the hamster, make sure they have food and water, and... my two dogs, they get taken out; then, I get ready for my day. I eat breakfast, take vitamins, make sure I have everything in my... bag for the rest of the day. I check my calendar because I am doing classes, research, work... so I have to make sure I have everything in my book bag for that” (Participant U8).

Participants also spoke of the importance of having a planning system. They described devising and using planning systems that centered primarily on classroom/academic demands. Most described using a planner or calendar to manage more than classroom assignments; they incorporated appointments and meetings related to their paid or volunteer work, career development activities (e.g., pre-professional clubs), and social life. Some even scheduled time specifically for daily living tasks as a way of ensuring that the time to meet personal goals was protected. One participant described, “I have a general life calendar that is all encompassing both personal and things I need to do” (Participant U9). Many participants also created a written plan for the week, month, semester, and for some, the year. Distant future-focused planning was used to prevent oversights regarding classwork and to ensure students remained on-track with the semester’s assignments, their course sequence, and their involvements with extracurricular career development activities.

Participants’ creation and use of prioritization strategies was important for effective use of the students’ devised planning systems. As shared by one participant, “As far as my homework, I usually do what’s due first and… if the task is like a really hard task, I’ll put that first also. So, the most important class, get that work done first” (Participant U30). Participants spoke of the importance of first determining which tasks are most urgent and then also strategizing as to which tasks might be able to serve multiple purposes across academic, daily life, and social contexts. For example, in order to spend time engaging in equally meaningful tasks, multiple participants spoke of spending time with friends by studying with them or socializing during their shared study breaks. A few participants referred to this strategy as “double dipping”. However, when unable to embed socialization into study time, participants described making decisions to decline or cancel previously scheduled social plans. The impact of such decision-making was described by one participant, “If I am scheduled to do something with someone and I have to cancel because I have something due the next day and I hadn’t bothered to do it until then, you know they’re frustrated and I am frustrated” (Participant U9).

Participants’ habits and routines were also supported through the use of reminder systems, which enabled many to ensure that planned tasks and activities were carried out in a timely manner. Multiple participants described a variety of reminder systems ranging from simple written checklists to electronic applications; only a few used mental checklists. Students spoke of relying on electronic platforms (i.e., apps), alarms, and even supportive others (e.g., roommates, friends) to remind them about tasks that needed to be completed and to help them stay on track for meeting their goals. One participant expressed, “I tend to think things through in the morning or the day and make like a list on my iPhone notes. It’s not the most organized method, but I organize my thoughts and determine what I need to accomplish during the day” (Participant U47). Several spoke of a preference for written daily lists. These participants described how the act of checking items off the list helped them to achieve a small but important feeling of accomplishment within their day. Additionally, participants who used written and/or mental checklists described the importance of finding the “just right” cue to include on the checklist. Some checklist descriptions could be too long, thus taking away from the ease in using the checklist. Decisions regarding the cue length needed to be balanced against ensuring that what was listed provided enough information to both prompt the task and to provide cues as to the critical aspects of the task demands. A few participants described less efficient reminder strategies. These participants spoke of logging into the web-based course management system throughout the day as a means of determining what assignments or study tasks needed to be worked on next. They spoke of systematically opening each course website in order to identify the most pressing task and then repeating the procedure multiple times throughout the day once a task was completed. Within the group discussions, participants were able to recognize the shortcomings of this strategy and were actively engaged in discussions of other strategies that were shared by other participants.

#### 3.2.2. Reframing

The reframing strategy was used by participants to better understand their temporal and productivity challenges. For participants, the process of reframing involved learning how to reframe challenging or frustrating experiences, such as not accomplishing a task or not managing one’s time as well as expected by self and others. The reframing strategy began with (1) self-evaluation, which then aided participants in (2) reframing their LD/ADHD challenges for both themselves and for others. The reframing strategy was used by participants as a means of protecting against the internalization of negative self-thoughts or perceived disappointment from others.

##### Reframing via Self-Evaluation

Self-evaluation involved techniques and supports used by the students to evaluate personal strengths and challenges and to better understand one’s learning style in order to direct development of personal, productivity and time-management goals and strategies. During the group meetings, this process was facilitated by focused questions that prompted the students to reflect and discover for themselves what worked and what critical aspects or situational conditions facilitated success. Participants not only shared their strategies, but during the guided discussions, most were able to identify which strategies were more successful, what could be done to improve their approach, and under what conditions the strategies were most effective. This process also facilitated the discovery of what skills or strategies needed additional refinement or even needed to be developed.

Evaluating strengths and challenges occurred when participants assessed areas in which they excelled while also identifying areas for growth. This was particularly meaningful when planning for their coursework as well as formulating study plans. Understanding one’s strengths and challenges was key in developing strategies to compensate for challenges related to academic coursework. For example, one participant shared, “[I’m] always going back to my strengths and weakness and figuring out okay, this class has a lot of vocabulary words in it, so it is going to be a little tougher. So, I am going to make flashcards, or I am going to have to create more mnemonics for this. [For this] class, I am going to have to work really hard because there is a lot of note taking. So, I am going to have to do a lot of reading outside of class and find some online resources for anything I [don’t understand]” (Participant U1). The appreciation of personal strengths and areas of weaknesses enabled participants to anticipate when they needed to allocate extra time to specific courses and thus manage their time and daily activities more effectively.

For study participants, evaluating personal strengths was intimately tied to an understanding of their preferred cognitive style and to a general understanding of different learning styles. Participants who were able to identify past learning situations that had worked well were guided to discern aspects of the situation that worked and then to compare those to aspects of their current successful situations. This enabled them to identify patterns in their learning strengths. It also enabled them to identify what works for them to increase efficiency when learning, as well as what works for them to minimize obstacles related to their specific learning processes.

Participants shared a variety of specific strategies based on their understanding of their preferred cognitive and/or learning style. For example, multiple participants identified the following as especially helpful: (1) recording lectures and listening to them as often as needed; (2) opting for online classes versus in-class courses when available, which enabled the students to view pre-recorded lectures multiple times; and (3) finding ways to conceptualize and organize tasks and activities in ways that allowed them to leverage big-picture thinking, which participants described as preferences for global and multidimensional thinking as opposed to detail-oriented or linear thinking. One participant described big-picture thinking and its impact in the following way: “I do get very impatient sometimes... especially when [I’m] thinking all these different things. You want something done real quick so I can go to the next thing... that takes a big-picture process, the connecting. I want to get to the next step as fast as I can, so I can actually see my web [of concepts and details] in [the] making” (Participant U29).

The self-evaluation strategy also required participants to reflect on challenges related to learning, task performance, and/or progress toward personal goals. For most, the process began by identifying an area or specific task in which improved performance was needed. As illustrated by one participant, “If I have something due, I may pull an all-nighter or wake up a couple hours early right before class... I don’t necessarily have the best study skills, but it is something I am working on” (Participant U9). For participants, understanding when improvement is necessary was the critical initial step for creating or identifying strategies to improve task performances. Such strategies most often encompassed those for improving task management, task organization, and the flow of the activity. Additionally, setting and monitoring small daily goals and then adjusting the goals as needed were important for facilitating incremental changes. Moreover, the use of small daily goals provided a structure that enabled participants to recognize positive effects of their efforts, which served as a motivator to keep them working toward desired changes. As shared, “Keeping a healthy routine as far as sleep isn’t generally something I have been good at. I am going to try again” (Participant U9).

##### Reframing for the Self and Others

Several participants needed to better accept their preferred approaches to tackling academic and daily challenges. They did this by discerning their personal preferences for solving everyday challenges and identifying the types of situations in which their preferred approach was a strength. In reframing their personal preferences regarding cognitive style (e.g., big-picture thinking) as a strength, participants articulated their strengths in solving challenges that (1) involved ambiguity, (2) required the brainstorming of multiple potential solutions, and (3) benefited from the quick identification of potential obstacles. In situations where these cognitive styles were not as beneficial, participants were supported in learning to recognize when they were achieving acceptable results despite inefficiencies in information processing and learning for that challenged task completion. For example, one participant reframed her learning process as follows: “When I am getting ready to solve a problem, if the teacher shows me one way and the class does it that one way, I don’t get it that way. I find [an]other way to do it, and my teacher will look at it and be like oh I see what you did. I just learn different” (Participant U46).

In reframing for oneself, participants were better able to self-advocate by reframing their LD/ADHD-related challenges for others. By being able to articulate their learning strengths and challenges, participants were better equipped to foster others’ awareness of and understanding of LD/ADHD. Several participants reported improved abilities in effectively speaking to instructors about which instructional practices best supported their learning needs. For example, participants were able to articulate their need for professors to leave material on the board longer or speak more slowly during lectures in order to compensate for difficulties in listening and simultaneously taking notes. Participants also expressed the understanding of potential universal benefits in advocating for the consideration of their learning styles and challenges. As expressed by one participant, “I also talk to my professors… ‘Hey, can you just leave it on the board for a little bit longer, some of the material?’… I mean that’s not… [just for] myself… but [for] other students I know who also complain about the same issue” (Participant U12). By using the reframing strategy and articulating their preferred learning style, participants were able to avoid spending precious time on becoming emotionally frustrated by others who did not understand their LD/ADHD-related needs. Reframing also enabled participants to avoid squandering time on attempting to solve problems or tackle academic tasks in ways that did not align with their preferred cognitive styles.

Additionally, some participants successfully reframed some of their LD/ADHD symptoms as a strength that could be extended beyond academic tasks; they were able to articulate the ways in which some symptoms or preferred cognitive styles could be helpful within certain types of work environments. “Just at work, I mean, I kind of feel like [LD/ADHD] makes me a better worker ‘cause I am always doing something. Like, I am not the kind of person that will sit around and be lazy... Most of the time, I am a server, and so, in a fast-paced restaurant, sometimes it helps to just be moving at a fast pace and try to do a million things at once” (Participant U45).

#### 3.2.3. Symptom-Specific Strategies

Symptom-specific strategies describe ways in which participants addressed specific LD/ADHD-related symptoms. Strategies were used to cope with challenges in sustaining focus and maintaining mental energy, memory, organization, and task initiation. Strategies included (1) planning activity breaks, (2) switching activities, (3) using environmental cues, and (4) creating low-level stress.

Participants described the importance of scheduling and taking breaks when working on academic assignments and focus-intensive tasks. “I make sure to give myself breaks while doing work, so I am able to be focused and not get distracted easily. I also make sure that I have an event or activity planned afterward, like hanging out with friends or playing basketball, so I am motivated to stay focused and work hard to complete my work” (Participant U38). This strategy was used as a way to refocus their thinking, to refresh the mental energy needed for task completion, and also to serve as a motivator. Participants also described using the Pomodoro technique, which is a time management method that breaks work tasks into intervals [26]. In describing the use of the Pomodoro technique, one participant shared, “Well, there’s like different ways you can do it. What I usually do is 25; what most people do is... four blocks of 25 min, and then in between, each one is a five-minute break. And then, after four blocks, you do a 25-min break, and then you continue doing them; after that... you’re supposed to get 11 to 12 done in one day. That’s usually my goal... After a while, you just kind of get used to it, and it’s a lot easier for me to work ‘cause sometimes when I sit down to do work, you get so much anxiety about having to think about sitting down for two hours and doing something for a long time. That’s what it helps me with” (Participant U48).

Activity switching is a strategy used to manage mental energy when studying. Most often, the activity switching strategy was used as a study break that enabled participants to maintain a productivity momentum by engaging in a task from another domain, such as household tasks. One participant stated, “I just can’t focus on one thing for a long time or else I would just go crazy... So, I would have [a] list, and I would just keep bouncing back and forth from one list to another because I feel like it’s going to be very productive for me. I feel like it’s better for me because I get more things done…” (Participant U46). Participants described keeping a written or mental list of tasks that can be quickly completed and required less mental energy than studying. These tasks ranged from household chores to easier academic tasks (e.g., homework or projects). At times, catching up with friends or going to the gym were used as ways to take mental breaks; these types of activities enabled the students to make progress toward their social and/or personal goals during the needed break.

Environmental cues are strategies used by participants to assist with challenges related to memory and leveraged strengths in visual processing. Participants strategically placed and/or arranged for environmental cues as a strategy for reminding themselves of tasks that needed to be completed or monitored; students described leaving visual cues around their living quarters and within their study spaces. They did this to assist with staying organized and to remind them to make time for things like household tasks. Others spoke of having friends and roommates serve as their reminders. “I usually set things up the day prior like if I have to do written online work done, I leave the windows open on my computer or I leave my work visible on my desk or if I am doing laundry I leave the hamper visible where it reminds me. ‘Cause, um, leaving notes I can’t, I won’t read them but if I visually see what’s going on, I can remember faster” (Participant U29). Environmental cues served as quick prompts that save time and mental energy by limiting the effort needed to plan things out.

Some students with difficulties in task initiation, focus, and/or motivation shared that for mentally challenging tasks, they work best and most quickly when under low-level stress. These participants spoke of intentionally using procrastination to create a low-level stress situation in order to fuel mental energy and/or enable the activation of hyper-focus for a task completion. “It’s, you know, one hour [left] and that’s it; like, I don’t have time to put it off [anymore], and the concentration that I get [from waiting to start] and the sort of effective work style that I get near an eminent deadline [is helpful] … and [working under a time pressure] that is replicated in a test environment” (Participant U9). Among the students who used procrastination as a driving force for completing academic tasks, several reported that having too much time as more stressful than the stress experienced during the urgency created by procrastinating. These participants spoke of the risks in accidentally over-procrastinating and not allocating enough time for task completion. Students who used this strategy risked inadvertently creating a level of anxiousness that prevented them from thinking clearly and effectively performing on the task. A few participants acknowledged that this strategy required them to balance their need for low-level stress against risks of emotional exhaustion, mental fatigue, and school burnout.

## 4. Discussion

This study was an in-depth examination of the strategies used to address time-related challenges to performance, and thus functioning and participation, experienced within the contexts of college students with disabilities’ everyday lives. We found that strategy use for addressing time-related and productivity challenges experienced by college students with LD/ADHD are multidimensional and involved far more than managing their calendars. Rather, time-related and productivity strategies used to support performance across life’s contexts entailed a mix of cognitive, behavioral, psychological, and socio-environmental strategies. The most-used strategies were those that supported performance beyond the students’ classroom contexts and had potential applications in future anticipated situations.

We found that participants needed to have an awareness of a range of potential strategies, an understanding of the types of situations that the strategies can be helpful in, and insights into the approaches for adjusting the strategies to meet specific situational demands. The presence of these factors was important for effective strategy use. Typically, strategies are automatically employed when handling a challenging situation [27]. However, our participants were benefited by the facilitated self-discovery/understanding of their strategy generation and application.

For individuals with LD/ADHD, interventions should include a focus on developing critical skills of strategy generation and tailoring, as well as the application of these strategies to new and varied situations and social contexts. Interventions should incorporate supports for guiding the individuals’ discovery of strategy generation, application, and modification. Guided discovery is one therapeutic approach that could be incorporated into such strategy training interventions. Guided discovery integrates processes of Socratic questioning (i.e., systematic and deep questioning) with a focus on problem-solving [28]. A variety of strategy training interventions have been successfully used with individuals from clinical populations experiencing impairments in cognitive functioning, such as autism [29] and traumatic brain injury [30,31]; similar interventions have also been successfully used with children with developmental coordination disorders to improve motor performance [32]. Research that tests the application of these established cognitive strategy training approaches with young people with LD/ADHD is warranted.

We found that effective strategy use across life’s contexts was critical to self-managing as a young person in college who is transitioning to adulthood with a chronic neurodevelopmental condition. This finding is consistent with research reporting the essential nature of executive skills to college success for those with LD/ADHD; critical executive skills include planning, goal setting, organization, flexibility, time management, and structuring time and tasks [23]. For our participants, the transition to adult roles involved simultaneously learning to cope with time and productivity challenges while also learning to self-manage LD/ADHD-related health, role, social, and emotional concerns.

The self-management of having a chronic condition requires core skills that include being able to effectively problem solve, garner supports, set appropriate goals and take action, and make adjustments when needed to action plans and strategies [33]. These skills can and should be taught long before a young person’s transition to adult roles and contexts. Interventions that work to improve children’s performances within the broad range of everyday life contexts are important for optimizing developmental trajectories and establishing skills that are foundational for effective self-management as an individual with LD/ADHD. The ability to carry out roles, routines, tasks, and subtasks for the purpose of meeting personal and societally ascribed expectations across life’s contexts is termed “occupational performance” and is the overarching goal of occupational therapy interventions [14]. Study findings illustrate the importance of multifaceted and multidisciplinary perspectives that include occupational therapy in treating LD/ADHD.

For our study participants, the fostering of (1) habits and daily routines that support organization and time management, (2) abilities for reframing and communicating LD/ADHD experiences, and (3) personalized understandings of LD/ADHD symptom manifestations and impacts, as well as understandings of personal strengths were key elements. These key elements were important for setting them up for success in coping with time-related and productivity challenges; they were key for facilitating their performance, functioning, and participation as college students learning to live as adults with a chronic neurodevelopmental condition. Moreover, these key supports facilitated participant’s abilities to meet performance expectations across the contexts of their everyday lives. As such, future studies should develop and test a manualized clinical rehabilitation (e.g., occupational therapy) intervention that provides these key elements in a systematic and patient-centered manner. Additionally, future research should investigate the relevance to of the strategies identified in this study to individuals with other clinical conditions whose neuropsychological profiles have similarities to those of individuals with LD and/or ADHD, such as college students with autism spectrum disorder.

Consistent with the study design of the secondary data analysis, data were not collected specifically to investigate strategy use for coping with LD/ADHD-related time and productivity challenges; thus, the findings provide only initial understandings of the key factors for supporting young people transitioning to adulthood through college. Ideally, these types of supports that foster occupational performance across life’s contexts are initiated during adolescence. The provision of such services would facilitate the transition to higher educational and work place settings. Future studies are needed that focus investigations on testing the processes and conditions for strategy development and application across everyday life contexts for school-age youth with LD/ADHD.

Our findings provide a much-needed understanding of the multifaceted challenges and solutions within young adult contexts that are important for guiding the development of interventions for young people with LD/ADHD. The findings provide insights that are vital for clinicians and parents in serving as an anticipatory guidance for use when making decisions about the care and support of children with LD/ADHD. Intentionally guiding young people in the generation, implementation, and adjustment of their strategies across the various contexts of everyday life is an important approach for improving their ability to meet the broad range of demands experienced in higher education and during the transition to adulthood. Interventions and supports for these young people should include supports for strategy and skill development (e.g., communication/self-advocacy skills) combined with the improved understanding of LD/ADHD-related challenges and personal strengths.

## Figures and Tables

**Table 1:** Participant demographics.

Gender *n* (%)	Race *n* (%)	Ethnicity *n* (%)
Male 26 (50) Female 24 (46) Not reported 2 (4)	White 37 (71) Black 8 (15) Asian 1 (2) Other 4 (8) Not reported 2 (4)	Hispanic 9 (17) Non-Hispanic 26 (50) Not reported 17 (33)

**Table 2:** Participant ratings of disability-related symptoms and challenges.

Symptom/Challenge *	Median ratings (*IQR*) ^∞^
Staying focused	75 (62, 94)
Managing time	65 (50, 81)
Extensive writing assignments	65 (31, 85)
Reading comprehension of textbooks or academic publications	64 (50, 81)
Organization	62 (47, 79)
Completing homework	56 (21, 73)
Memorizing and retrieving information from memory	57 (23, 85)
Following multistep directions ^β^	56 (34, 70)
Expressing thoughts or opinions clearly	53 (22, 71)
Following others when they speak in conversation	50 (21, 73)
Applying different approaches to one problem	38 (18, 56)
Initiating activities, tasks, or independent ideas	34 (18, 63)

*, *n* = 51; ∞, Ratings reported using a digital analog scale from 0 (never) to 100 (always); β, *n* = 50; *IQR*, Interquartile range.

**Table 3:** Strategies for addressing time-related and productivity challenges.

Strategy Category	Definition	Strategy Subtypes
Habits and routines	Strategies that students used to organize and plan their time (e.g., day, week, semester, and distant future)	Highly structured and productive morning routine Planning systems Prioritization Reminder systems
Reframing	Strategies used to redefine disability-related challenges to increase personal understanding which can be used to explain LD/ADHD-related challenges to others	Self-evaluation of (1) strengths and challenges, (2) learning style, and (3) goals Reframing for the self and others
Symptom-specific strategies	Strategies used to cope with specific LD/ADHD-related symptoms	Planning activity breaks Activity switching Environmental cues Creating low-level stress
